# The stability of articulatory and acoustic oscillatory signals derived from speecha)

**DOI:** 10.1121/10.0036389

**Published:** 2025-04-18

**Authors:** Jessica Campbell, Dani Byrd, Louis Goldstein

**Affiliations:** Department of Linguistics, University of Southern California, Los Angeles, California 90089, USA jessica.campbell@usc.edu, dbyrd@usc.edu, louisgol@usc.edu

## Abstract

Articulatory underpinnings of periodicities in the speech signal are unclear beyond a general alternation of vocal tract opening and closing. This study evaluates a modulatory articulatory signal that captures instantaneous change in vocal tract posture and its relation with two acoustic oscillatory signals, comparing stabilities to the progression of vowel and stressed vowel onsets. Modulatory signals can be calculated more efficiently than labeling linguistic events. These signals were more stable in periodicity than acoustic vowel onsets and not different from stressed vowel onsets, suggesting that an articulatory modulation function can provide a useful method for indexing foundational periodicities in speech without tedious annotation.

## 1. Introduction

Relatively slow oscillations in acoustic speech signals have been evidenced in speech signal processing using various filters and transforms (see review in Ref. 1) with the goal of identifying low-dimensional information in speech.^2^ In the clinical realm, the variability of these low-dimensional signals may reveal evidence of excessive or limited variability in production for speakers with motor speech disorders. These signals are especially useful for indexing regularities or periodicities in speech, as they do not require the laborious annotation of linguistic events. However, the causal source of acoustic periodicity—namely, the modulation of vocal tract articulation—has not been investigated beyond the lips and jaw^3,4^ (see, e.g., Smith^5^) We evaluate a derived articulatory signal that captures modulatory change in vocal tract posture and assess its relation to two acoustic oscillatory signals, comparing their relative stabilities in reference to the acoustic progression of vowel onsets (VOs) and stressed vowel onsets (SVOs). This approach to obtaining speech periodicities can be calculated with more ease, uniformity, and computational efficiency than can approaches that label linguistic events. Such a method is especially useful in the clinical domain, where it may be used to quantitatively index breakdown in speech motor control.

A low-dimensional signal that captures the oscillatory properties of articulation is the spatiotemporal articulatory modulation function—the AMF—first introduced in Goldstein.^6^ Unlike the spatiotemporal index (STI),^7^ which provides a single score across repetitions of an utterance, the AMF is a dynamic signal over time and is calculated across one instance of an utterance. The AMF captures the oscillatory nature of the actions of the vocal tract by calculating a profile of instantaneous change in vocal tract posture, incorporating multiple articulators so as to index global articulatory change or kinetic energy over time. Calculation of the AMF is relatively easy to implement and efficient to calculate with articulatory data in hand.

The AMF is calculated as the sum of the squared Euclidean distance between successive time samples for points or surfaces along the vocal tract (we use seven points; see Poeppel and Assaneo's^8^ brief mention of a similar function). Local peaks in this function have been dubbed “modulation pulses” (MPs).^6^ MPs are discrete events in the dynamic modulation time function. Studies of AMFs have analyzed the frequency of these pulses.^9^ (That said, magnitude of the pulses may prove of interest in future work.) This current report assesses periodicity and stability in the AMF in comparison with two low-dimensional acoustic modulation functions: spectral flow (SF) and the acoustic amplitude envelope (AAE). Such spectral and amplitude functions have been well-investigated; however, their causal basis in articulation has not, apart from action of the jaw with respect to amplitude variation.^3^ Articulatory flow, via the AMF or similar signals, has been largely overlooked (though see two recent considerations in Refs. 10 and 11).

Just as the AMF captures postural change in the vocal tract, spectral modulation captures similar change in the acoustic domain. In particular, SF sums the squared change in mel-frequency cepstral coefficient (MFCC) parameters over time, thereby capturing oscillatory properties of global spectral change.^6,12,13^ The AMF and SF have been shown to be correlated and phase-locked.^6,14^

The AAE is the most thoroughly explored low-dimensional characterization of modulatory patterns in spoken language. It captures slow-moving amplitude oscillations of speech, generally reflecting alternations between high-amplitude vowels and low-amplitude consonants at the rough granularity of the syllable.^15^ It has been observed that peaks in change in the AAE are time-locked with neural firing patterns in the superior temporal gyrus,^16^ though some top-down predictive mechanisms may play a role.^17^

The present study will test the stability of AMF, SF, and the AAE (we use the derivative of the traditional AAE^15^) comparing the variability of their frequencies to the variability of the frequencies of syllables (generally) and stressed syllables (specifically). The goal of this investigation is to validate an index of periodicity using these modulatory signals that is more efficient and systematic to calculate than is measuring durations between linguistic events, which is the traditional method of quantifying temporal regularities in speech.

## 2. Method

### 2.1 Participants and material

Data were sourced from the publicly available X-ray Microbeam (XRMB) corpus,^18^ which includes data from 57 American English speakers who read utterances of various types while the positions of small gold markers adhered to the surface of key articulators were tracked over time. The material analyzed included readings of the first section of the *“Grandfather Passage.”*^19^ To ensure sufficiently fluent data, the speakers who were included were those who completed this with no disfluencies and at most ten mistracked or missing data points (which were replaced by interpolation).^20^ Nine speakers fit these criteria and were used in the analysis.

The XRMB corpus includes the acoustic signal of the read speech sampled at 21 739 Hz and synchronized with a two-dimensional (2-D) position time series of markers on the mandible, upper lip, lower lip, ventral tongue, mid-tongue, and dorsal tongue, which were resampled at 145.65 Hz. For the present study, speech was analyzed from the first speech-specific articulatory movement in the passage up to a predetermined intonational phrase boundary occurring after the passage clause *“his voice quivers a bit,”* ending at the completion of the tongue tip gesture for the final [t] in *“bit.”* Each participant's audio was segmented into syllables as well as phonemes and pauses using the Penn Phonetics Lab Forced Aligner (Penn Phonetics Laboratory, University of Pennsylvania, Philadelphia, PA).^21^

### 2.2 Calculations of low-dimensional characterizations of speech

A low-dimensional articulatory modulation function was calculated to profile instantaneous change in the vocal tract over time, following Goldstein.^6^ Specifically, to generate the AMF, the squared Euclidean distance between the vector of XRMB marker positions at successive time samples was calculated and then smoothed using a 12 Hz lowpass ninth-order Butterworth filter.

SF was calculated similarly as change in the first 13 MFCC parameters over time, excluding the first (see likewise Goldstein^6^). The squared Euclidean distance was calculated in the 12-dimensional space across each audio time sample and then smoothed using a 12 Hz lowpass ninth-order Butterworth filter.

The AAE was calculated following Tilsen and Arvaniti^15^ and Campbell *et al.*^9^ Analysis intervals (see below) were individually filtered using a [400, 4000] Hz cutoff Butterworth filter to remove high-amplitude frication and then normalized, followed by the application of a moving Tukey window (*r* = 0.2). To yield the final AAE, the time series was differentiated to determine change over time and then filtered with a 12 Hz lowpass fifth-order Butterworth filter to parallel the process of calculating AMF and SF.

**Fig. 1.**
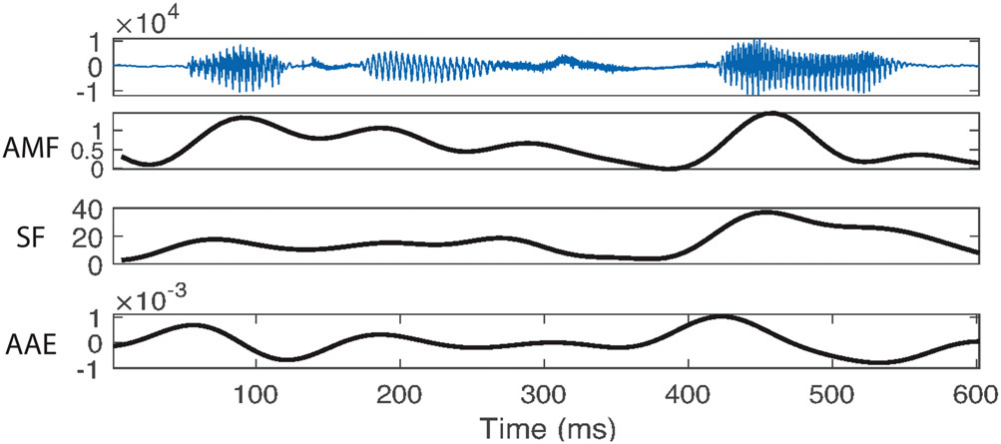
Three modulatory time signals for the phrase *“yet he still”* (participant 39).

Peaks in the AMF, SF, and AAE were calculated by locating local maxima in the functions (Fig. 1). This was implemented by shifting the function over by one sample in both directions and locating the times at which the original function showed a higher magnitude than the shifted functions. Following Goldstein,^6^ peaks in these modulatory signals are called “pulses.” The frame rate was 145.6452 Hz for the AMF and the SF, with the SF frames based on 25 ms analysis windows. The Butterworth (cutoff value) filter used to calculate the AAE was calculated on all the samples in each analysis interval, which varied based on produced intonational phrases (average of 2.83 s), and gave the same number of samples as in the original audio (sample rate = 21 739 Hz).

### 2.3 Analysis intervals

Analysis intervals for the signals were chosen based on linguistic phrasal groupings. Stretches of speech between consecutive potential intonational phrase boundaries in the passage, based on orthographic commas and periods, were identified as possible analysis intervals. However, not every speaker produced a pause at every potential phrase boundary. Rather, analysis intervals were only segmented at phrase boundaries that were produced with a pause of 100 ms or longer. Thus, the number of analysis intervals per speaker varied depending on how many pauses they produced. Analysis intervals were not affected by pauses due to disfluencies (see Sec. 2.7). Only modulation pulses occurring within an interval so defined were included in the quantitative analyses. AMF and SF were calculated prior to chunking the signals into analysis intervals, while the AAE was calculated over the individual analysis intervals.

### 2.4 Syllable annotation

The synchronized audio of each participant's passage reading was linguistically annotated by the experimenter (author 1) using praat. Each syllable in the passage was annotated as having either primary stress or not—labeled “stressed” vs “unstressed” (with the latter category including lesser secondary stresses). The Carnegie Mellon University Pronouncing Dictionary^22^ was consulted in rare cases of ambiguity. Monosyllabic function words were considered unstressed, while monosyllabic content words were considered stressed. For the planned analysis of syllable and stressed syllable frequencies, the acoustic onset of the nucleus of each syllable (generally a vowel) was defined as the syllable's timepoint of occurrence and was manually corrected when necessary after applying the Penn Phonetics Lab Forced Aligner,^21^ placed at: the waveform zero-crossing closest to the start of the formant transitions in cases following sonorants or silence, at the stop burst before aspiration for vowels following stops, or at the beginning of voicing in the waveform for vowels following fricatives.

### 2.5 Derivation of dependent variables

Within each speech analysis interval, frequency means and distribution were calculated for five oscillatory time series: (i) pulse frequency in the AMF, (ii) SF pulse frequency, (iii) AAE pulse frequency, (iv) syllable frequency (VOs), and (v) stressed syllable frequency (SVOs; roughly correspondent to linguistic “stress feet”). Recall that time series (iv) and (v) were determined by manual annotation. Frequency was calculated from the periods between pulses or vowel onsets, and the reciprocal of these periods provided the frequencies for each time series.

The variation or stability of time series (i) through (v) was calculated both within and between speakers. To quantify and compare within-speaker variation, the coefficient of variation (CV) was calculated as the standard deviation (SD) divided by the mean. The process for quantifying and comparing between-speaker variation consisted of comparing mean-normalized squared residuals of linear mixed models that included random intercepts grouped by speaker.

### 2.6 Statistical analyses

To compare the variabilities of the three low-dimensional oscillatory time series—AMF, SF, and AAE—within each speaker with the variabilities of the progression of VOs and SVOs, differences between CVs were tested using six modified signed-likelihood ratio tests (MSLRTs) for equality of CVs per speaker.^23,24^ (The pseudorandomization seed was arbitrarily set to 95.) Six tests were conducted for each speaker; these compared the frequencies of the three oscillatory functions (AMF, SF, and AAE) to the two vowel onset functions (VO and SVO)—specifically (i) AMF vs VO, (ii) SF vs VO, and (iii) AAE vs VO and (iv) AMF vs SVO, (v) SF vs SVO, and (vi) AAE vs SVO. Bonferroni corrections were applied to these 54 pairwise comparisons, with a resulting *p <* 0.00093 considered significant.

To compare the variabilities of each time series across speakers, mixed effects models for pulse and onset frequencies of all five time series functions (AMF, SF, AAE, VO, and SVO) were fitted individually on all speakers' pooled data using the r package *lme4*, predicting the frequencies of each time series only by random intercepts grouped by speaker. Residuals from these models thus only represent the variation in intercept between the speakers. The residuals from each model were normalized by squaring and dividing by the mean frequencies of all speakers pooled. The normalized residuals were then compared between the modulatory signals and the linguistic events in six new linear models that predicted normalized residuals by time series, specifically (i) AMF vs VO, (ii) SF vs VO, (iii) AAE vs VO, (iv) AMF vs SVO, (v) SF vs SVO, and (vi) AAE vs SVO. In these models, the presence of significantly higher normalized residuals for one time series vs another indicates that that time series has significantly greater between-speaker variability relative to the other.

### 2.7 Data exclusion

While analysis intervals were segmented by pauses at intonational phrase boundaries, some participants produced (ungrammatical) filled or silent pauses that did not occur at phrase boundaries. These pauses, especially when silent, typically featured few pulses, which could have affected the variability calculation of the frequencies for each time series. For this reason, periods between pulses or onsets with at least one edge occurring during pauses longer than 100 ms (as marked by the Penn Phonetics Lab Forced Aligner^21^) were excluded.^25,26^

## 3. Results

### 3.1 General observations

The low-dimensional oscillatory signals were qualitatively periodic for all speakers. The mean pulse frequencies with speakers pooled were as follows: AMF frequency 8.18 Hz (SD = 2.31), SF 9.25 Hz (SD = 3.14), and AAE 7.22 Hz (SD = 2.45). Table 1 presents these results by speaker, along with results for SVOs and VOs, which uniformly have lower frequencies.

**Table 1.** Average pulse frequencies of each time series for each speaker.

Speaker ID	AMF frequency		SF frequency		AAE frequency		VO frequency		SVO frequency	
	Mean (Hz)	SD	Mean (Hz)	SD	Mean (Hz)	SD	Mean (Hz)	SD	Mean (Hz)	SD
28	7.97	2.51	9.08	2.96	7.46	2.37	5.26	2.58	2.38	1.06
39	8.43	2.29	9.66	2.94	7.03	2.68	6.57	4.75	2.84	1.26
41	8.49	2.50	8.83	2.76	7.65	2.24	7.36	5.27	3.21	2.05
43	7.76	2.06	9.38	4.54	6.97	2.24	6.44	6.02	2.64	1.48
46	8.25	2.17	9.22	3.47	7.15	2.65	6.47	4.29	2.86	1.26
48	7.76	2.40	9.33	2.79	7.31	2.73	5.84	4.92	2.51	1.03
51	8.30	2.22	9.12	2.56	6.95	2.33	6.80	5.06	3.30	2.52
53	8.35	2.30	9.16	2.76	7.06	2.59	6.01	2.60	2.76	1.16
61	8.30	2.25	9.40	2.99	7.27	2.18	6.21	3.61	2.57	0.93

### 3.2 Analysis of regularity

**Fig. 2.**
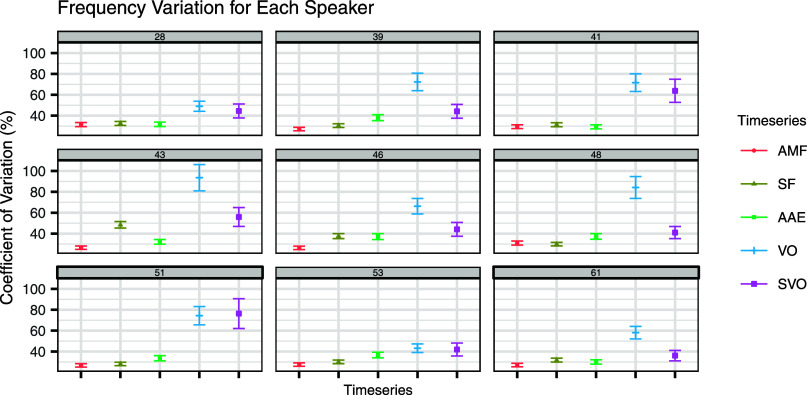
Variability of frequencies for each speaker. Panel numbers represent speaker ID. Error bars indicate the standard error of the CV.

To assess variability within each speaker, the CVs for the pulse frequencies of each modulation function and each vowel onset series were calculated for each speaker. (Because AMF, SF, and AAE typically have higher frequencies than SVOs, CV is preferred over SD for this assessment.) The results across speakers were then averaged. The mean CVs across speakers were 28.14% for AMF pulses, 33.36% for SF pulses, 33.99% for AAE pulses, 68.06% for VOs, and 49.77% for SVOs. CV results for each speaker are presented in Fig. 2.^27^

To assess whether these variability differences were significant, MSLRTs for equality of CV^23^ were conducted for each speaker. When compared to VO frequency, all speakers showed significantly lower variability in both AMF pulse frequency (*p* < 0.0001 for all speakers) and in SF pulse frequency (*p* < 0.001 for all speakers). All speakers but one showed lower variability in AAE pulse frequency than VO frequency (*p* = 0.18 for speaker 53; *p* < 0.0002 for all others). Results were less consistent when comparing the three modulatory signals to the SVO frequency. Four out of nine speakers showed significantly lower variability in AMF pulse frequency than in SVO frequency, two showed lower variability in SF pulse frequency than in SVO frequency, and three showed lower variability in AAE pulse frequency than SVO frequency. Table 2 details each speaker's Bonferroni-corrected statistical results.

**Table 2.** Variability comparison of frequencies of each time series.^a^

	Articulatory Modulation Pulse Frequency		Spectral Modulation Pulse Frequency		Differentiated Amplitude Envelope Pulse Frequency	
	vs VO Frequencies	vs SVO Frequencies	vs VO Frequencies	vs SVO Frequencies	vs VO Frequencies	vs SVO Frequencies
Speaker ID	MSLRT	MSLRT	MSLRT	MSLRT	MSLRT	MSLRT
28	15.52^*^	4.93	13.71^*^	3.97	14.02^*^	4.50
39	69.70^*^	10.72	56.51^*^	5.94	22.84^*^	0.63
41	55.04^*^	25.72^*^	48.05^*^	21.41^*^	50.27^*^	24.65^*^
43	103.51^*^	26.27^*^	24.11^*^	0.53	61.92^*^	12.03^*^
46	62.63^*^	12.14^*^	21.87^*^	0.79	19.06^*^	0.88
48	66.38^*^	3.16	81.04^*^	4.30	36.65^*^	0.23
51	75.11^*^	50.11^*^	70.83^*^	45.94^*^	35.50^*^	23.79^*^
53	17.30^*^	7.96	11.12^*^	4.68	1.78	0.51
61	46.51^*^	3.49	29.41^*^	0.54	29.69^*^	1.20

^a^ Bonferroni-corrected *p* < 0.000 93.

**Fig. 3.**
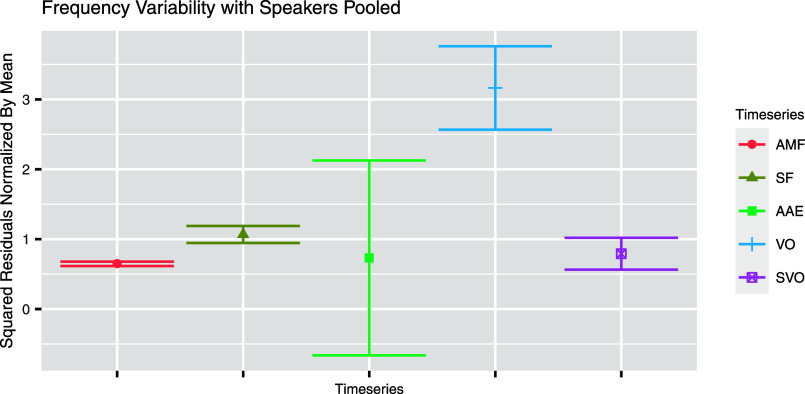
Normalized squared residuals for each time series. Error bars represent the standard error of the residuals.

To examine between-speaker variability, an analysis of residuals of linear models was employed (Table 3). The frequency data from all speakers were pooled for each of the five time series. A linear model was fitted for each data set, predicting frequency by only random intercepts grouped by speaker and no traditional predictor; this allowed for the comparison of variability across speakers while still accounting for within-speaker variation. (For SF, the resulting model was singular, indicating insufficient difference between speakers' mean frequencies, so the random intercept was removed.) For example, squared residuals from the AMF pulse model were divided by the mean AMF frequency across speakers; these were then compared, using a linear model predicting residuals by time series, to the normalized squared residuals from the VO model. A significant difference in residuals between the two time series would represent a significant difference in between-speaker variability for AMF vs VO (Fig. 3).

**Table 3.** Linear model results for time series comparisons.^a^

Articulatory Modulation Pulse Frequency							
vs VO Frequencies				vs Stressed VO Frequencies			
Coefficient	*⁁β*	SE(⁁*β*)	*t*	Coefficient	*⁁β*	SE(⁁*β*)	*t*
Intercept	0.65	0.24	2.66^*^	Intercept	0.65	0.05	12.66^*^
Time series	2.52	0.42	5.96^*^	Time series	0.15	0.12	1.17
*R^2^* = 0.02, residual SE = 9.03 (*df* = 2062), *n* = 2064				*R^2^* = 0.0008, residual SE = 1.90 (*df* = 1660), *n* = 1662			

Spectral Flow Pulse Frequency							
vs VO Frequencies				vs Stressed VO Frequencies			
Coefficient	*⁁β*	SE(⁁*β*)	*t*	Coefficient	*⁁β*	SE(⁁*β*)	*t*
Intercept	1.07	0.24	4.42^*^	Intercept	1.07	0.12	9.00^*^
Time series	2.10	0.44	4.81^*^	Time series	−0.27	0.30	−0.91
*R^2^* = 0.01, residual SE = 9.51 (*df* = 2235), *n* = 2237				*R^2^* = 0.0005, residual SE = 4.67 (*df* = 1833), *n* = 1835			

Differentiated Amplitude Envelope Pulse Frequency							
vs VO Frequencies				vs Stressed VO Frequencies			
Coefficient	*⁁β*	SE(⁁*β*)	*t*	Coefficient	*⁁β*	SE(⁁*β*)	*t*
Intercept	0.73	0.30	2.48	Intercept	0.73	0.06	11.33^*^
Time series	2.43	0.48	5.12^*^	Time series	0.06	0.14	0.42
*R^2^* = 0.01, residual SE = 9.74 (*df* = 1772), *n* = 1774				*R^2^* = 0.0001, residual SE = 2.13 (*df* = 1370), *n* = 1372			

^a^ ^*^p < 0.05.

These models found significantly lower variability between speakers for AMF pulse frequencies as compared to VO frequencies [*β* = 2.52, standard error (SE)(*β*) = 0.42, *t*(2062) = 5.96, *p* < 0.001], between speakers for SF pulse frequencies as compared to VO frequencies [*β* = 2.10, SE(*β*) = 0.44, *t*(2235) = 4.81, *p* < 0.001], and between speakers for AAE pulse frequencies as compared to VO frequencies [*β* = 2.43, SE(*β*) = 0.48, *t*(1772) = 5.12, *p* < 0.001]. There was no significant difference between speakers for SVO frequencies and AMF pulse frequencies [*β* = 0.15, SE(*β*) = 0.12, *t*(1660) = 1.17, *p* = 0.24], nor for SF pulse frequencies and SVO frequencies [*β* = −0.27, SE(*β*) = 0.30, *t*(1833) = −0.91, *p* = 0.36] or AAE pulse frequencies and SVO frequencies [*β* = 0.06, SE(*β*) = 0.14, *t*(1370) = 0.42, *p* = 0.67].

To summarize, AMF, SF, and AAE pulses exhibited greater stability in frequency than VOs both within and between speakers. In contrast, all three low-dimensional characterizations of speech generally exhibited comparable stability to SVOs, though some individual speakers did exhibit greater stability for one or more of the three low-dimensional modulatory signals than for SVOs.

## 4. Discussion

This study examines three low-dimensional indices of speech oscillation with respect to the regularity of syllables and stressed syllables for English. In general, all three low-dimensional characterizations of speech oscillation behaved similarly to one another when compared to progression of VOs and SVOs. Overwhelmingly, the AMF and its correlated SF function, as well as the differentiated AAE, were found to be less variable in frequency than acoustic VOs both within and between speakers. In contrast, the three signals were found to be comparable in regularity with SVOs, even though dynamic changes in global vocal tract posture, spectral properties, and amplitude generally occur at higher frequencies than does the progression of stressed syllables. Thus, these efficient and replicable modulatory signals could be used to index regularity or stability in speech, potentially in place of indices requiring the labeling of linguistic events such as syllables in English. This method of indexing foundational periodicities in speech could be used to analyze both linguistic rhythmic properties and breakdown of speech in specific clinical populations. Last, the comparable results observed for all three oscillatory signals validate the utility of acoustic modulation signals for indexing patterns of articulatory modulation. Thus, the SF and AAE may be confidently understood to reflect temporal regularity in the production domain in research or clinical settings without access to articulatory data. In sum, this study demonstrates that the acoustic oscillatory signals could be used as a proxy for the modulation patterns of the causal articulatory signal, in situations where the collection of articulatory data is difficult, expensive, or unavailable.

### 4.1 Limitations of this study

The results of the present study provide new information regarding the frequency and stability of several periodicities in speech production. Nonetheless, these findings should be contextualized within several limitations. Specifically, production of stressed syllables may not always match canonical lexical entries. Annotating stress in this study was based on the canonical lexical entry for each word, but it is possible that speakers did not always produce a pronunciation following this canonical form. Second, our protocol for determining vowel onset timepoints used specific criteria that could be altered. Third, our results only address read, English speech without disfluent repetitions. Last, our AMF relied on kinematic point-tracking data that were informative only about the oral, upper-vocal tract articulators, lacking vocal tract movement information for the velum, larynx, and pharynx.

### 4.2 Potential utility

The ease with which global speech production stability can be indexed using low-dimensional modulatory signals suggests its worthwhile application to domains of linguistic and clinical analyses. Relative variability in the modulatory properties of the speech signal could provide a new characterization of cross-linguistic rhythmic properties in languages both with and without predictable stress. Additionally, quantification of variability of these modulatory signals could be used as an accessible and replicable metric in assessment of speech dysfunction. Many speech disorders such as dysarthrias in neurodegenerative diseases are associated with prosodic deviations from typical speech.^28^ The variability of AMF, SF, and AAE pulses could index this rhythmic breakdown without requiring laborious annotation of interval durations, thereby increasing efficiency and systematicity.

## 5. Conclusion

In summary, the AMF captures low-dimensional oscillatory properties of instantaneous change in vocal tract posture in the speech production domain. It behaves similarly to previously identified SF and differentiated AAE acoustic periodicities when compared to the regularity of syllables and stressed syllables in English. The periodicities of all three low-dimensional signals—articulatory, spectral, and amplitude—exhibit greater stability than the periodicity of overall syllable progression but generally exhibit stability comparable to that of stressed syllable progression. In conclusion, we establish low-dimensional modulatory functions derived from speech as a useful and efficient method for indexing foundational periodicities in both the articulatory and acoustic domains of speech without the need for tedious linguistic annotation.

## Supplementary material

See the supplementary material for details of mixed linear models for each time series in the between-speaker variability analysis.

## Data Availability

Raw data were generated at the University of Wisconsin X-ray Microbeam large scale facility. Derived data supporting the findings of this study are available from the corresponding author upon reasonable request.
